# Analysis of a Splice Array Experiment Elucidates Roles of Chromatin Elongation Factor Spt4–5 in Splicing

**DOI:** 10.1371/journal.pcbi.0010039

**Published:** 2005-09-16

**Authors:** Yuanyuan Xiao, Yee H Yang, Todd A Burckin, Lily Shiue, Grant A Hartzog, Mark R Segal

**Affiliations:** 1 Department of Epidemiology and Biostatistics, Center for Bioinformatics and Molecular Biostatistics, University of California, San Francisco, California, United States of America; 2 Department of Medicine, Center for Bioinformatics and Molecular Biostatistics, University of California, San Francisco, California, United States of America; 3 Department of Molecular, Cell, and Developmental Biology, University of California, Santa Cruz, California, United States of America; 4 Department of Molecular, Cell and Developmental Biology, University of California, Santa Cruz, California, United States of America; University of California, San Diego, United States of America.

## Abstract

Splicing is an important process for regulation of gene expression in eukaryotes, and it has important functional links to other steps of gene expression. Two examples of these linkages include Ceg1, a component of the mRNA capping enzyme, and the chromatin elongation factors Spt4–5, both of which have recently been shown to play a role in the normal splicing of several genes in the yeast *Saccharomyces cerevisiae*. Using a genomic approach to characterize the roles of Spt4–5 in splicing, we used splicing-sensitive DNA microarrays to identify specific sets of genes that are mis-spliced in *ceg1, spt4,* and *spt5* mutants. In the context of a complex, nested, experimental design featuring 22 dye-swap array hybridizations, comprising both biological and technical replicates, we applied five appropriate statistical models for assessing differential expression between wild-type and the mutants. To refine selection of differential expression genes, we then used a robust model-synthesizing approach, Differential Expression via Distance Synthesis, to integrate all five models. The resultant list of differentially expressed genes was then further analyzed with regard to select attributes: we found that highly transcribed genes with long introns were most sensitive to *spt* mutations. QPCR confirmation of differential expression was established for the limited number of genes evaluated. In this paper, we showcase splicing array technology, as well as powerful, yet general, statistical methodology for assessing differential expression, in the context of a real, complex experimental design. Our results suggest that the Spt4–Spt5 complex may help coordinate splicing with transcription under conditions that present kinetic challenges to spliceosome assembly or function.

## Synopsis

Splicing is a key process for the regulation of gene expression in eukaryotes and is credited as being the main reason for the extraordinary complexity of the human proteome relative to the human genome. Accurate splicing is crucial for normal protein function; aberrant transcripts due to splicing mutations are known causes for 15% of genetic diseases. Therefore, elucidation of splicing mechanisms will not only help in understanding the complexity and diversity of higher organisms, but also potentially aid in new therapeutic strategies for treatments of splicing-related genetic disorders. It has been previously shown that splicing has important links to other steps involved with gene expression. In this study, the authors pursue a genome-wide approach, using yeast-based, splicing-sensitive, DNA microarrays in order to further characterize the roles of select splicing factors. They devise novel statistical and computational methods that enable identification of specific sets of genes that are mis-spliced in the chosen splicing factors. Follow-up investigation of known attributes of the genes so elicited indicates that these factors may help coordinate splicing and transcription in situations where additional energy is required to effect splicing.

## Introduction

Eukaryotic genes are fragmented into exons by intervening sequences (introns). After a gene is transcribed into pre-mRNA, the introns are removed from the transcript and the exons are joined by the spliceosome. This reaction, splicing, can also be used to create multiple transcripts from a single gene. For example, a particular exon may be included in one version of an mRNA, and skipped in another. This process of alternative splicing is subject to regulation in response to tissue, developmental, and environmental cues [1]. In humans, most genes are subject to splicing and more than half are likely subject to alternative splicing, which is credited as the most important source for the extraordinary enrichment in complexity of the human proteome relative to the genome [1]. Accurate splicing is crucial for normal protein function; aberrant transcripts due to splicing mutations are known causes for 15% of genetic diseases [1]. Therefore, elucidation of splicing mechanisms will not only help us understand the operating mechanisms underneath the functional complexity and diversity of higher eukaryotes, but also aid in new therapeutic strategies for treatments in splicing-related genetic disorders.

Although the different steps of gene expression are typically studied in isolation, it is clear that there are important functional links between them [2–4]. For example, the process of capping the 5′ end of pre-mRNAs is thought to influence both transcription and splicing [5,6]. Furthermore, the rate of transcription elongation appears to influence splicing and alternative splice site choice [7,8]. In addition, a number of pre-mRNA processing factors are recruited to transcripts via interaction with RNA polymerase II [2,3]. Thus, a comprehensive description of mRNA synthesis will require an understanding between these functional linkages of steps in gene expression.

Traditionally, gene expression is studied on an individual gene basis by ad hoc experiments. With the advent of eukaryotic genomic sequences, a global genomic view of mRNA production is achievable, and recently, several large-scale gene expression profiling experiments utilizing microarray technology have provided an unprecedented amount of information regarding the mechanisms underlying its regulation [4,9–11]. *Saccharomyces cerevisiae*, a simple yeast that has been used as a model to study eukaryotic gene expression, presents a convincing entry point to embark on this task. The yeast genome is completely sequenced and well annotated, and the splicing machinery of yeast is well conserved with that of humans. Among the more than ~5,800 genes in the yeast genome, only about 250 of them possess introns and only a handful have multiple introns or are alternatively spliced [12]. However, these 250 intronic genes give rise to 27% of the transcripts synthesized by the cell, an indication of the importance of splicing in yeast [13,14].

**Figure 1:**
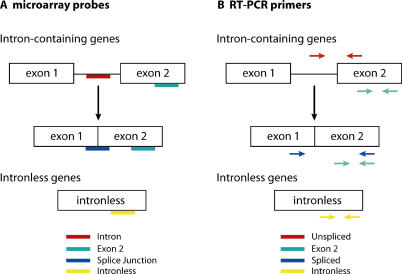
Splicing Array Probe and RT-PCR Primer Design (A) Probe design of the splicing array. There are three oligonucleotide probes for each intron-containing gene: intron (red), splice-junction (blue), and exon (green). In addition, there are also about 800 probes for intronless genes (yellow). This figure is modified from Clark et al. [15]. (B) Primer design of RT-PCR. Primers are chosen to flank the intron–exon2 junction and the second exon or spliced mRNA.

Clark et al. [15] designed a DNA microarray that allows the simultaneous analysis of splicing and mRNA levels in yeast. To discriminate between spliced and unspliced transcripts, oligonucleotide probes on these arrays were designed to detect the splice junctions (SJ), introns, and second exons of intron-containing genes (Figure 1A). SJ are found only in spliced transcripts, whereas introns exist only in unspliced transcripts and splicing intermediates. Second exons are present in both spliced and unspliced transcripts and are good indicators of total transcript level. To detect these different classes of transcripts, the arrays are competitively hybridized with probes derived from control and experimental yeast strains. For several splicing mutants, Clark et al. compared their whole genome splicing data to traditional molecular analyses of a small number of transcripts and found that reliance on only one or two genes as reporters may lead to misinterpretation of the role of a factor in splicing [15]. Thus, the whole genome approach provides a more reliable method for assessing the role of particular factors in splicing.

Burckin et al. [4] recently used splicing-sensitive DNA microarrays to analyze 80 different yeast strains carrying mutations in genes encoding components of the gene expression machinery. Using clustering and machine learning techniques, they compared gene expression patterns in these mutants and discovered functional roles for specific factors at multiple steps in the gene expression pathway, further confirming the coordination and coupling of the machineries along the pathway.

Previously, Hartzog et al. [16] found evidence that the chromatin elongation factors Spt4 and Spt5 play a role in RNA processing in *S. cerevisiae*. Spt4 and Spt5 form a complex that regulates transcription elongation by RNA polymerase II. This complex is conserved across eukaryotes and has been proposed to both facilitate transcription by removing a nucleosomal barrier to transcript elongation and also suppress inappropriate transcription by reassembling nucleosomes behind transcribing polymerase [16]. The recent finding that Spt5 interacts physically and genetically with pre-mRNA capping factors suggests a role for Spt4–Spt5 in capping [17–20]. Because pre-mRNA capping is thought to increase the efficiency of splicing, Lindstrom et al. further analyzed splicing in *spt4* and *spt5* mutants and found that several genes were not spliced with normal efficiency [17]. In the splicing array study described above, Burckin et al. [4] found extensive but not universal splicing defects in *spt4* and *spt5* mutants. Interestingly, they also found that the capping enzyme appears to play an essential role in splicing. Thus, their genome-wide analysis of splicing provided particularly striking examples of linkages between steps in gene expression. However, the experimental design of that study precluded identification of specific genes dependent upon particular factors for their splicing.

Such identification is the purpose of our present study. While we also utilize splicing-specific DNA microarrays, we do so in the context of an experimental design that enables elicitation of specific intron-containing genes that are mis-spliced in *spt4, spt5,* or *ceg1* mutants. In addition, we examine the aberrant splicing patterns caused by several phenotypically distinct *spt5* mutations that had not been previously examined. Our primary data analytic task is therefore the determination of the set (possibly empty) of genes that have altered expression as reported by the SJ and intron probes. To do this, we assayed each mutant multiple times and then employed a recently devised statistical framework that robustly and efficiently identifies genes exhibiting differential expression (DE) in the mutants.

Many methods have been advanced for this task of identifying differentially expressed genes. Fold change has been extensively used to yield lists of genes that have altered expression beyond a prescribed threshold. Despite its methodological simplicity and intuitive appeal, fold change lacks a statistical framework (there is no accommodation of expression variation) and is biased toward selecting genes at low expression levels. Another class of frequently used methods treats the task of comparing expression levels in different biological states as a univariate testing problem, employing various corrections for test multiplicity [21]. Kerr et al. [22] propose using traditional analysis of variance (ANOVA) techniques, since these readily handle known sources of variation due to, for example, dye labeling and sample or array replicates. By removing these effects from the estimation of the error term, we achieve a reduction in this term and correspondingly sharper inferences. Wolfinger et al. [23] extend the ANOVA framework by treating some factors, for example, dyes and arrays, as random representatives of a large population (that is, as random effects) resulting in a mixed model. There are several Bayesian alternatives to the above approaches [24–27], as well as some intermediary approaches that yield regularized *t* statistics [28–30].

Our study employs a complex experiment design, featuring 22 dye-swap array hybridizations comprising both biological and technical replications (see Results). As elaborated in the next section, we initially analyzed these data with four ANOVA mixed models and the semiparametric hierarchical mixture model (SHMM) of Newton et al. [31]. Instead of arbitrating between these models and picking a single model on which to base DE declarations, we exploit the fact that all five models are estimating the same quantity and employ a novel synthesizing scheme [32], Differential Expression via Distance Synthesis (DEDS), to derive a list of differentially expressed genes in *spt* mutants. This method compares favorably with the best individual models, while enjoying improved robustness properties [32]. Further analysis of such genes, whose splicing is altered in *spt* mutants, reveals common biochemical characteristics and attributes, which may provide new insights into the mechanisms of RNA processing and its connections to transcription.

## Results

### Experimental Design and Data Pre-Processing

In yeast, *SPT4* is a non-essential gene encoding a 102-amino-acid protein, and *spt4Δ* (null) mutants display mutant phenotypes and genetic interactions consistent with an elongation defect [16]. *SPT5* encodes a large protein, and *spt5* mutations typically display mutant phenotypes and genetic interactions similar to those observed for *spt4* mutations, although they are often phenotypically more severe, consistent with the fact that *SPT5* is essential for life [16]. In this work, we have analyzed an *spt4* null mutation, and three partial loss-of-function mutations in *SPT5*. Two of these, *spt5–4* and *spt5–194,* encode versions of Spt5 that are defective for binding Spt4 (GAH, J. Yamada, and T. Egelhofer, unpublished data). The third allele, *spt5–242,* causes a cold-sensitive growth defect [33], and displays splicing and other defects at all temperatures (GAH and TB, unpublished data; [17]). The Spt5–242 protein still binds Spt4, even at the non-permissive temperature (GAH, J. Yamada, and T. Egelhofer, unpublished data). In addition, we include analysis of *ceg1–250,* a temperature-sensitive mutation that causes rapid inactivation of the capping enzyme at the non-permissive temperature [6].

**Figure 2:**
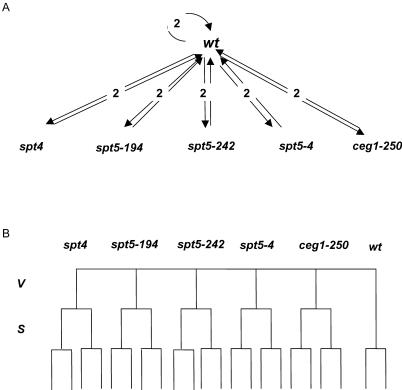
Graphical Representation of Designs (A) In this representation, vertices correspond to target mRNA samples and edges to hybridizations between two samples. By convention, we place the green-labeled sample at the tail and the red-labeled sample at the head of the arrow. (B) Nested design of the experiment. The effect *A* is nested in *S,* and *S* is in turn nested in *V*. Note that there are two samples *(S)* for each mutant, but only one sample for the wild-type.

Two independent mRNA samples were prepared from each mutant, fluorescently labeled, and then hybridized to the splicing arrays competitively with a probe derived from wild-type cells. Experiments were performed using a replicated dye-swap study design (Figure 2A) [34]. Briefly, there were four arrays (A1–A4) for each mutant versus wild-type experiment. The first mRNA sample was hybridized to arrays A1 and A2 (Figure 2B), and the second was hybridized to A3 and A4. In A1 and A3, the mutant mRNA sample was labeled with Cy5 dye, and the wild-type sample was labeled with Cy3. The dye assignment was reversed for arrays A2 and A4. In addition to these 20 mutant arrays (four arrays × five mutants), there were two separate wild-type self-hybridization experiments, in which the wild-type was labeled with both Cy5 and Cy3. These self-hybridizations serve as technical replicates, that is, as controls for variation in labeling and hybridization.

**Figure 3:**
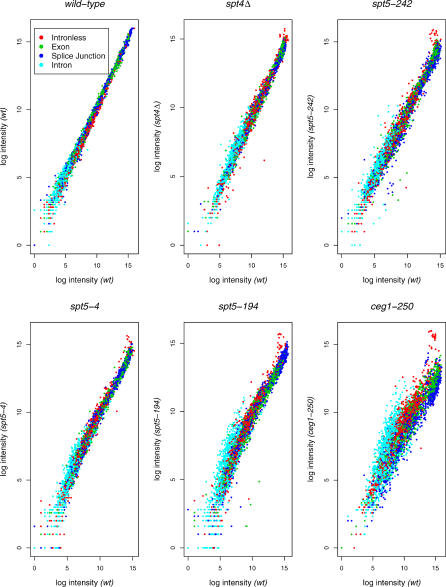
Scatterplots of the Logarithm Intensities of Splicing-Related Probes Points are color-coded as indicated.

To provide a global view of splicing defects in the *ceg* and *spt* mutants, we plotted unnormalized log intensity values for signals from the two channels, mutant against wild-type, in Figure 3. Points that represent individual array features are color coded so that exon, SJ, intron, and intronless gene features can be visually differentiated. Genes lying on the diagonal have a ratio close to 1, and their expression in the mutants is therefore largely unaffected. For *ceg1–250,* shown in the lower right panel, introns (light blue points) deviate noticeably from the diagonal toward the *ceg1–250* axis. This is a clear indication of intron accumulation in the *ceg1* mutant. SJ (dark blue points) in *ceg1–250,* on the other hand, largely display ratios of less than 1, indicating a decrease in SJ formation. Taken together, an accumulation of introns and loss of SJ in *ceg1–250* is indicative of a splicing defect. Compared with *ceg1–250*, the four *spt* mutants exhibit fewer alterations in splicing, with *spt5–194* most severely affected, in agreement with its phenotypic characteristics. A control plot from the wild-type self-hybridization is depicted in the upper left panel. As expected, no separation is observed in introns and SJ, and all points conform closely to the diagonal.

**Figure 4:**
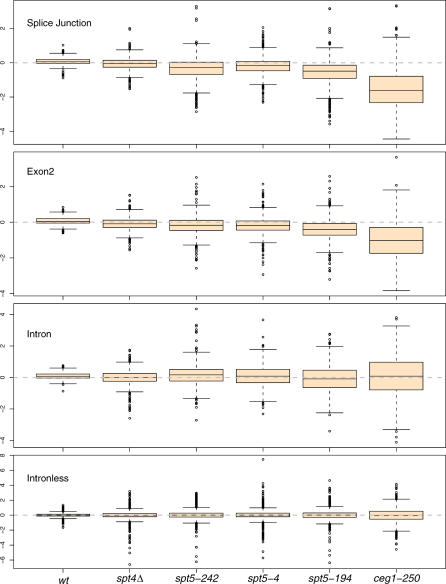
Boxplots of Normalized Ratios of Splicing-Related Probes Stratified by Mutants Splice-junction and exon probe ratios show a shift from the horizontal zero line in the negative direction, whereas intron probe ratios are centered at zero.

**Figure 5:**
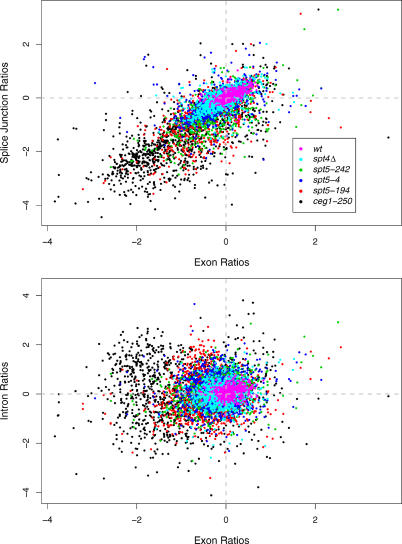
Scatterplots of Normalized Ratios of Splicing-Related Probes Points are color-coded by their mutant identity. Gray horizontal and vertical reference lines indicate zero expression ratios.

Boxplots of normalized ratios of splicing-related probes stratified by mutants are shown in Figure 4. The general trend of the SJ probe ratios shows a shift from the horizontal zero line in the negative direction, signaling a decreased expression of SJ in the mutants. The *ceg1–250* mutant showed the largest decrease, and *spt5–194* was the most severely affected of the *spt* mutants. The boxplots of the exon probe ratios display a similar pattern of change—the expression of exon probes was also decreased in the mutants. This is consistent with the idea that the majority of the exon 2 probe signal for a transcript is derived from mRNA, which is stable and long-lived in comparison to pre-mRNA. It is of interest to investigate if the decrease of the SJ probe and exon probe ratios is correlated. Figure 5 displays the scatterplots between ratios of these probes. The upper panel shows evident correlation between SJ and exon ratios. In contrast to the exons and SJ, ratios of the intron probes do not show any shift from the horizontal zero line, but spread for the mutants is nonetheless increased. Furthermore, there is no obvious correlation between the intron and exon ratios. In both plots, however, the spread of the cloud of points is mutant dependent and related to the severity of splicing defects. From Figure 4, it is clear that several of the mutants tested, *ceg1–250, spt5–194,* and *spt5–242,* cause strong decreases in exon and SJ signals and, more idiosyncratic, gene-specific changes in intron signals. Do these changes reflect altered transcription, splicing, RNA decay, or a mixture of potential defects? To focus on alterations of splicing efficiency independent of changes in transcription, we used the intron accumulation (IA) and SJ indices of Clark et al. [15], which normalize ratios of intron and SJ signals to the ratios measured for the second exon. The SJ index is the change of the SJ probe signal normalized by the change of overall gene expression level as measured by the related exon probe signal: *SJ* = log(*SpliceJunction_mut_*/*SpliceJunction_wt_*)/(*Exon_mut_*/*Exon_wt_*). Similarly, the IA index is obtained as the normalized change of the intron probe signals: *IA* = (*Intron_mut_*/*Intron_wt_*)/(*Exon_mut_*/*Exon_wt_*). Relating changes in the SJ and intron signals to changes in the second exon takes into account changes in overall expression level that may occur as a result of alterations in other steps of gene expression.

### DE Models

The experimental design of the splice mutant study motivated the use of four different mixed ANOVA models in addition to the SHMM (Table 1). These were separately applied to the two splicing indices. The four ANOVA models are distinguished by including wild-type self-hybridizations or not and allowing gene-specific heterogeneity or not. The experimental design (Figure 2B)—wherein array effect *A* is nested in sample effect *S (S/A),* and sample effect *S* is in turn nested in mutant effect *V (V/S),* argues for treating model terms involving *S* and *A* as random effects, with the remaining terms involving genes *(G),* mutants *(V),* and gene–mutant interactions *(GV)* being fixed effects. The four models thereby constitute nested, mixed-effect ANOVAs ; see Materials and Methods for fitting details.

**Table 1:**
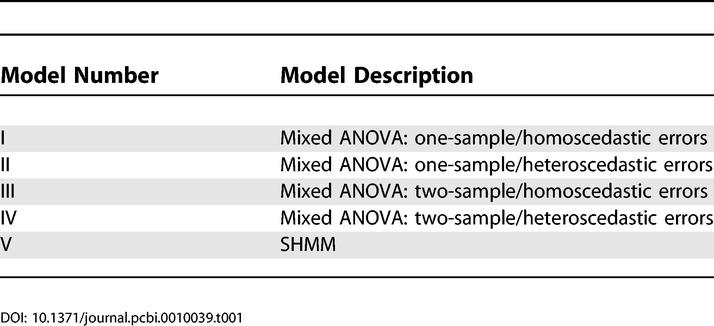
Summary of the Five Competing DE Models

To complement the ANOVA approaches described above, we also employed the SHMM advanced by Newton et al. [31]. This methodology was selected for the following reasons: (i) the SHMM is non-parametric where there is sufficient information (lots of genes) and parametric where there is limited information (observations per gene), and this synthesis makes for an appropriately balanced strategy; and (ii) as is standard, our ANOVA approaches treat gene *(G),* mutant *(V),* and gene–mutant interactions *(GV)* as fixed effects. Thus, there is no information sharing between genes. The SHMM achieves such sharing and does so in a more principled and flexible manner than some of the ad hoc approaches proposed that yield regularized *t*-statistics [28,30,35]. The SHMM also has limitations, the foremost of which perhaps is the adequacy of the parametric assumptions. The extent of such assumptions has been appreciably relaxed compared to the preceding fully parametric treatment of Kendziorski et al. [27]. Importantly, diagnostic tools are provided for assumption checking. Additionally, the present implementation supports only two group comparisons. Thus, there is some potential efficiency loss for the nested design employed in the splice study (Figure 2B). Details on the estimation methodology as well as extensive illustration of calibration, diagnostic, and performance aspects are provided in Newton et al. [31].

### Model Synthesis and Selection of Differentially Expressed Genes

Models with heteroscedastic errors accommodate gene-specific variances, but typically, as here, replication is very limited and so the precision of the estimates is compromised. Models imposing homoscedastic errors yield precise estimates of the common error variance, and tests based on many degrees of freedom (df), since they permit combination over the large number of genes. However, the homoscedasticity assumption is both strong and difficult to evaluate. Differences in error df for the different models are presented in Table 2. Note that there are more than 5,000 df for error for the homoscedastic models and only about 20 df for the heteroscedastic models. A comparison of associated *p*-values from individual measures is provided in [32].

**Table 2:**
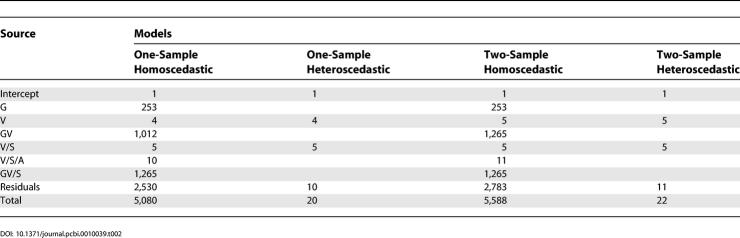
Degrees of Freedom for the ANOVA Mixed Models

DEDS is a novel method combining statistics or summaries that measure the same phenomenon [32]. Rather than trying to arbitrate between models and pick a single model on which to base DE declarations, or informally distilling sets of genes that are differentially expressed under two or more models, we applied DEDS here as a robust means to refine selection of DE genes as furnished by the above five individual models. The simple underlying principle of DEDS is that genes that are highly ranked (as being differentially expressed) by all five models are more likely to be truly differentially expressed than genes that are high only for a single model. Further details concerning DEDS are provided in [32], while an algorithm outline is sketched in Materials and Methods.

**Figure 6:**
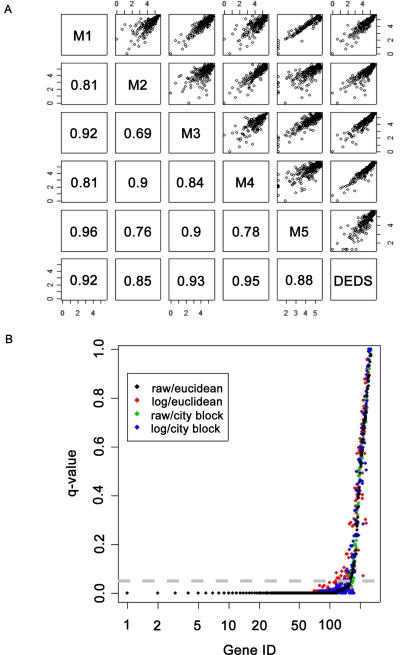
Analysis of DE Gene Ranking and Selection by DEDS (A) A comparison of ranking of DE genes by DEDS and individual measures. Plotted are a scatterplot matrix of DE gene rankings by the five models and DEDS using *spt5–242* IA indices. Ranks are logged so that correlations of DE genes (low ranks) are more clearly displayed. (B) Sensitivity of DEDS declarations of DE to choice of scale and distance metric. Genes, ordered according to their DE significance by the raw/Euclidean combination (so that the black points are monotone by definition) are plotted against DEDS *q*-values for all four scale/distance combinations. The dashed gray line marks the 0.05 *q*-value threshold.

The task of identifying differentially expressed genes consists of two components: (i) ranking genes in order of evidence for DE; and (ii) declaration of a set of DE genes by thresholding the ranked list. Here, we examine the robustness of DEDS with respect to both components. A comparison of ranking of DE genes by DEDS and individual measures, using an example based on the IA indices of *spt5–242,* is provided in Figure 6A. Ranks are logged so that correlations of DE genes (low ranks) are more clearly displayed. We see a similar level of concordance between ranks from DEDS and individual models. The numbers of genes identified as differentially expressed by DEDS under false discovery rates (FDR) 0.01 and 0.05 for SJ as well as IA indices are listed in Table 3. To examine the stability of the DE findings, we assessed the impact of scale choice of measures and the interrelated choice of distance metric. We investigated all combinations of (raw, logarithm) *p*-value scales * (Euclidean, city block) distances. Representative results are shown in Figure 6B. Genes are ordered according to their DE significance by the raw/Euclidean combination, so that the black points are monotone by definition. The dashed gray line marks the 0.05 *q*-value threshold. The corresponding numbers of declared DE genes for these four combinations range from 145 to 163, of which 139 are common to all four combinations. This demonstrates that, for the splice array experiment, DEDS-based selections of DE genes are largely insensitive to the different scales and distance metrics examined. This concordance, evident in Figure 6, pertains to the IA index and the *ceg1–250* mutant. Analogous concordance was observed for the SJ index and the other mutants.

**Table 3:**
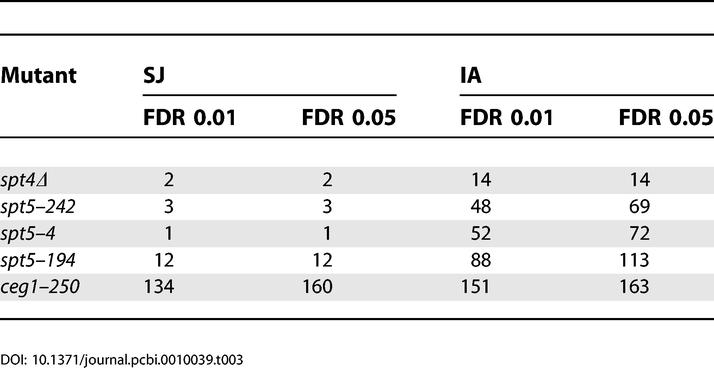
Number of DE in SJ and IA Indices

The observation of greater numbers of genes identified as differentially expressed based on the IA index data than on the SJ data (Table 3) reinforced the finding in Clark et al. [15] that IA indices are a more sensitive indicator for splicing defects. The splicing defect in the yeast capping enzyme mutant *ceg1–250* is catastrophic, whereas in the *spt4* and *spt5* mutants fewer genes exhibit a splicing defect. Overall, *spt5–194* is the most severe splicing mutant among all *spt* mutants, with *spt4Δ* being the least impaired. The complete list of DE genes is provided in Table S1.

### Validation of DE Genes

The identification of genes affected by *spt4* and *spt5* mutations using statistically robust methodology offers insight into the function of the Spt4–Spt5 complex, as well as the opportunity to better equate changes in IA with bona fide splicing defects. To validate our findings, we have used quantitative RT-PCR (QPCR) analysis to quantitatively examine five intron-containing genes, as well as two unspliced genes, in all five mutants. We previously performed a qualitative analysis of three of these genes, *U3B, RPS25A,* and *RPL26A,* and found that they were inefficiently spliced in *spt4* and *spt5* mutants [17]. By choosing primers that flank the intron–exon2 junction, we can specifically detect unspliced pre-mRNA (Figure 1B). We also picked primers to detect either the second exon, or spliced mRNA (Figure 1B). As with the microarrays, we can normalize changes in pre-mRNA levels to changes in spliced mRNA or total mRNA (that is, exon2).

As shown in Table 4, the results of the RT-PCR analysis generally agreed with the microarray analysis. Strikingly, in the four *spt* mutants, genes identified by DEDS showed an absolute increase in pre-mRNA levels, while in the *ceg1* mutant none of the pre-mRNAs showed an absolute increase as compared to wild-type. After normalizing the pre-mRNA signals to the spliced mRNA or second exon signals to account for potential changes in transcription or transcript stability, *ceg1* also showed a splicing defect as predicted by DEDS. Furthermore, the performance of DEDS was superior to the four ANOVA models and equivalent to the SHMM in terms of numbers of false positives and negatives over all five mutants (Table S2).

**Table 4:**
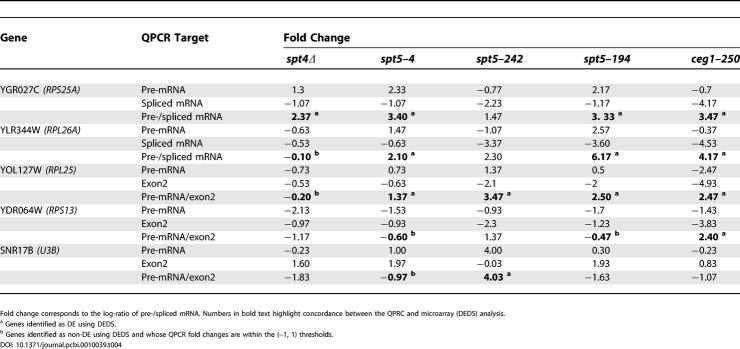
QPCR Validation DE Microarray Data

### Description and Analysis of DE Genes

There are likely multiple molecular mechanisms by which different genes were differentially expressed in the mutants discussed here. To account for some of these mechanisms, we subdivided the lists of DE genes with a *q* ≤ 0.05 (controlling FDR) before further analysis. First, we reasoned that positive and negative changes in IA likely occurred via different molecular mechanisms. Therefore, for each of the five mutants examined, the DE genes were divided into lists of genes with either positive or negative fold change (here, fold change refers to the IA index). Second, because ribosomal protein genes represent a large fraction of all spliced genes in yeast [36], and because they are subject to a common mode of regulation [37], we further subdivided our lists of DE genes into sublists of ribosomal (RP) and non-RP genes (Table 5). Finally, we focused upon the IA index, as it is more sensitive to alterations in splicing [15].

**Table 5:**
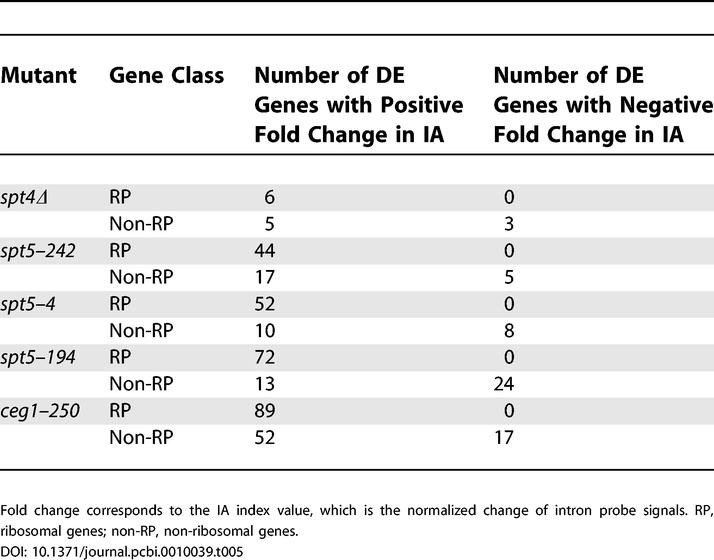
Distribution of DE Genes

For the *spt5* and *ceg1* mutants, a large majority of the DE genes encoded RP proteins, whereas only ~40% of all intron-containing genes encode RP proteins (Table 5 and [36]). Furthermore, a number of translation and rRNA processing factors are among the non-RP genes found in our analysis, and it is possible that these genes are regulated by the same strategies as the RPs. Interestingly, for those DE genes with a negative fold change—that is, those that were apparently spliced more efficiently—we found no RP genes. This suggests that the genes with a negative or positive fold change in the IA index have distinct dependencies upon Spt4–Spt5 and Ceg1.

We next asked if the genes identified in this analysis shared any particular attributes. It has previously been noted that introns in yeast display a bimodal distribution of sizes and positions within genes [36]. RP protein genes have large introns that occur relatively early in a pre-mRNA, whereas non-RP genes typically have smaller introns that occur somewhat later in the mRNA. Furthermore, RP genes are highly transcribed, whereas non-RP genes tend to be less highly transcribed [14]. We therefore compared the transcription rates and size and positions of introns within the DE genes that displayed a positive fold change (Table 6). In the *ceg1* mutant, the set of DE genes had no unusual properties other than the non-RP DE genes being transcribed somewhat more frequently than the average non-RP gene. In the *spt* mutants, intron position of the DE genes was not significantly different from the average for RP and non-RP genes (Table 6). In contrast, in the *spt5–4* and *spt5–194* mutants, the non-RP DE genes shared attributes of RP genes: they tended to have longer introns and be more highly expressed than the typical non-RP gene. The non-RP DE genes in the *spt4Δ* and *spt5–242* mutants represent an intermediate case; their introns are not significantly longer than those of the typical non-RP intron-containing genes, but they are more highly transcribed.

**Table 6:**
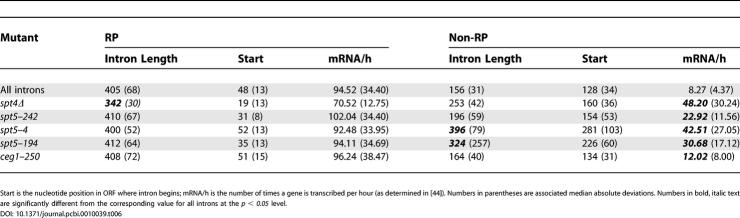
Properties of DE Genes with a Positive Fold Change (Average)

The DE genes with a negative fold change appear to represent a distinct class of genes. First, they encoded only non-RPs. Second, they resembled the typical non-RP intron-containing genes in that they had short introns; however, they were expressed at even lower levels than the typical non-RPs (Table 7), contrary to the DE genes with positive fold changes. Again, this is consistent with the idea that these genes were differentially expressed for reasons distinct from those leading to DE of genes with a positive fold change.

**Table 7:**
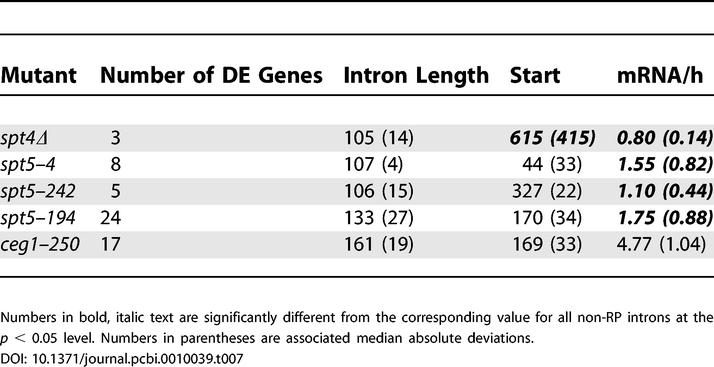
Properties of DE Genes with a Negative Fold Change (Average)

## Discussion

In this paper, we showcased splicing array technology and developed methodologies for its analysis in the context of a real, complex experimental design. We applied four ANOVA mixed models and a SHMM, and used DEDS [32] to derive a list of DE genes. The DEDS algorithm synthesizes statistics or methods that estimate the same quantity of interest. The underlying principle behind DEDS is that genes that are highly ranked by different methods are more likely to be truly differentially expressed than genes that rank highly on a single measure. In our previous work, we have evaluated DEDS on diverse datasets, featuring both one-channel Affymetrix oligonucleotide arrays and two-channel spotted arrays [32]. Using a set of spike-in (Affymetrix) datasets, where differentially expressed genes are known, we demonstrated that DEDS compares favorably with the best individual statistics while enjoying robustness properties lacked by the individual statistics [32].

**Figure 7:**
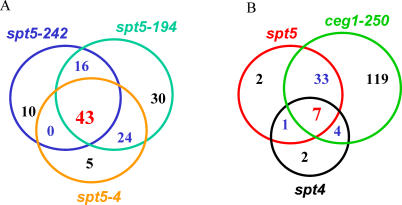
Venn Diagram of DE Genes from Different Mutants (A) compares DE genes among the three *spt5* mutants *(spt5–194, spt5–4,* and *spt5–242).* Statistical test shows that the common 43 genes are highly significant, with a *p*-value *<* 0.001. In (B), *spt5* refers to the 43 common genes among all *spt5* mutants. The overlaps between *spt5* and *ceg1–250* (40, *p <* 0.001), *spt5,* and *spt4* (8, *p<* 0.001), *spt4*, *spt5,* and *ceg1–250* (7, *p<* 0.001) are all significant.

Previous to this and other microarray studies, only four genes had been identified and confirmed for splicing defects in *spt4* and *spt5* mutants using traditional molecular techniques [17]. Recently, Burckin et al. have used splicing-sensitive DNA microarrays to compare patterns of splicing defects across a diverse set of mutations affecting gene expression [4], but this and the previous study lacked a statistical or quantitative framework for rigorous determination of specific genes that were differentially expressed. Here, we have used splicing-sensitive DNA microarrays combined with DEDS to analyze all known intron-containing genes in the yeast genome and to specifically identify those genes whose proper splicing is dependent upon *SPT4, SPT5,* or *CEG1*. Despite the differences in experimental goals and designs, the findings of these two studies are nonetheless consistent. In Burckin et al. [4], the analyses using hierarchical clustering and support vector machines showed that the overall impact of the loss of Ceg1 function in vivo is nearly identical to that of bona fide splicing factors, which is in line with the large number of DE genes we found whose splicing is abnormal in *ceg1–250* (see Table 3). Also in our previous study, *spt4Δ* and *spt5–194* mutants displayed a lesser splicing defect than the *ceg1–250* mutant, which is again consistent with our current findings. Comparison of the lists of DE genes for the five mutants examined here revealed that most of the genes that were differentially expressed in the *spt* mutants were also differentially expressed in the *ceg1* mutant (Figure 7 and Table S1). The *spt5–242* mutant differed from the other *spt5* mutants in that it did not preferentially affect the splicing of non-RP genes with long introns. We do not understand the mechanistic basis for this observation, although it is consistent with our previous observations that this *spt5* mutation is phenotypically distinct from other *spt5* alleles and therefore may cause a distinct biochemical defect [33,38]. Our data further suggest that Spt4′s contribution to splicing is modest, as only a handful of genes were differentially expressed in the *spt4* mutant. This is consistent with the observation that, in contrast to *SPT5, SPT4* is not essential for life. Furthermore, this observation suggests that the defects caused by the *spt5–4* and *spt5–194* mutations extend beyond the Spt4 binding defect we have observed for the Spt5–4 and Spt5–194 proteins. Since there is currently no evidence that Spt4 functions independently of Spt5 [39], these observations suggest that Spt4 assists in, but is not essential for, the functions of Spt4–Spt5 in splicing.

The smaller number of DE genes in the *spt* mutants compared to *ceg1–250* may indicate a lesser effect on splicing rather than an effect on a distinct subset of intron-containing genes. It is interesting to note, however, that highly transcribed genes with long introns—that is, RP genes and a subset of non-RP genes with long introns—were most sensitive to the *spt* mutations. These data suggest that the Spt4–Spt5 complex may play a particular role in coordinating splicing with transcription under conditions that present kinetic challenges to the spliceosome or its assembly, that is, when splice sites are widely separated, increasing the separation in time and space between the synthesis of the 5′ and 3′ splice sites, or when a gene is highly transcribed, creating the need for rapid and repeated assembly of spliceosomes over one site on a gene. In addition, these data are consistent with recent evidence demonstrating an effect of RNA polymerase II elongation rates on alternative splicing in higher eukaryotes [40]. In contrast, the non-RP genes spliced more efficiently in the *spt* mutants tend to be transcribed less frequently than the average non-RP gene (Table 7). Thus, as is the case for transcription, the Spt4–Spt5 complex may have both positive and negative effects on splicing [16]. Furthermore, this is consistent with previous observations that altered transcription elongation may lead to increased splicing, presumably due to increased opportunities for recognition of suboptimal splice sites [7,8]. Whether the effects we have measured here are due to altered elongation rates, or they indicate a more direct role of Spt4–Spt5 in splicing is currently under investigation.

## Materials and Methods

### Sample preparation and array hybridization.

All yeast strains (Table 8) used were isogenic to S288C and Gal+ [41]. Yeast were grown overnight in rich medium (YPD) at 30 °C to early log phase (> 1 × 10^7^ cells/ml), spun down, and resuspended in pre-warmed 39 °C media, and allowed to grow at 39 °C for 45 min after shift to restrictive temperature. Cells were collected by centrifugation at room temperature for 4 min, washed once with sterile water, flash frozen in liquid nitrogen, and stored at −80 °C. Total RNA was isolated by a hot phenol method [42] and quantitated by UV absorbance. Fluorescently labeled probe preparation, hybridization, and data acquisition were performed as previously described [15] using 15 μg of total RNA/sample. For each mutant, RNA was prepared from two independently grown cultures. Each RNA sample was used to probe two arrays, and was labeled with Cy3 for the first array and Cy5 for the second.

**Table 8:**
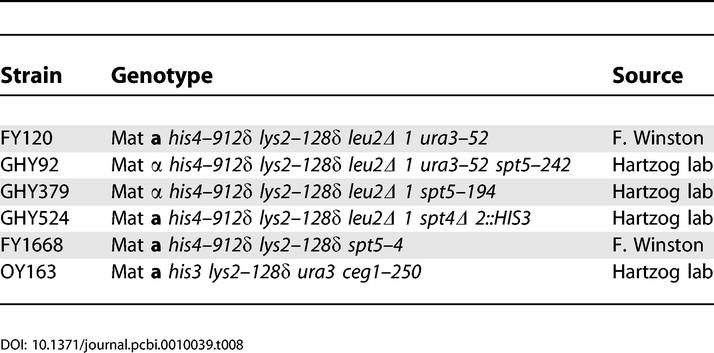
Yeast Strains

### Data normalization and pre-processing.

To effectively and properly normalize the data, we used non-linear *loess* normalization [43] based on the subset of intronless genes. After normalization, for each array the four replicates of each SJ, intron, and exon probes were summarized using averages. This was followed by the calculation of SJ and IA indices.

### ANOVA mixed models.

We applied four different ANOVA mixed models corresponding to all combinations of wild-type versus wild-type (in/out) by gene-specific variance heterogeneity (yes/no). DE is examined by the two-sample *t* test, when including the two wild-type versus wild-type samples, whereas a one-sample *t* test is applied when excluding these two samples. Not allowing for gene-specific variance imposes the assumption that all genes exhibit a similar degree of variability, so they can be jointly analyzed using a common estimate of error variance [22]. Conversely, allowing different variances for different genes [23] mandates fitting gene by gene.

### Model specifics.

Model I—one-sample/homoscedastic errors: Let *Y_gvsa_* be the splicing related index, SJ or IA, from gene *g* (*g = 1, 2,…, 254* for SJ and *1, 2,…, 263* for IA), mutant *v (v = 1, 2,…, 5),* sample *s (s = 1,2),* and array *a* (*a = 1,2;* corresponding to the dye-swap pair). The first model can be represented as


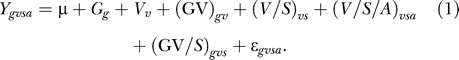


**Table 9:**
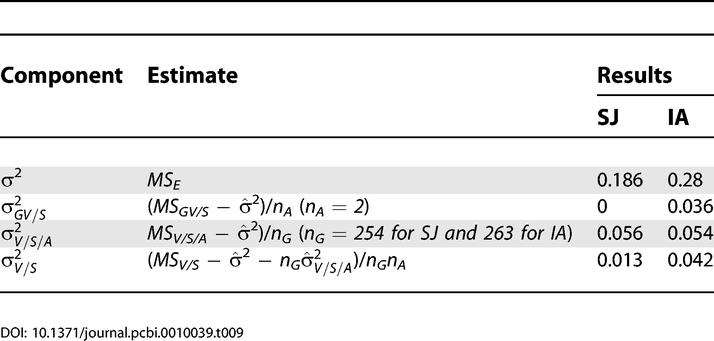
Derivation of Variance Components for Model I

Effects *(V/S)_s_, (V/S/A)_a_, (GV/S)_gvs_,* and ɛ_*gvsa*_ are assumed to be normally distributed normal variables with zero means and variance components 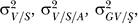 *,* and σ^2^, respectively. The derivation of the variance components is shown in Table 9. The remaining effects in the model are fixed effects. The parameter of interest in this model is *μ_gv_* = *μ* + *G_g_* + *V_v_* + *GV_gv_*, which measures the mean of the SJ/IA indices of gene *g* in mutant *v*. The following null hypothesis therefore defines the absence of DE in mutant *v* and gene *g*:


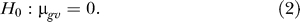


The variance of the treatment mean μ̂_*gv*_ can be computed by the following equation:


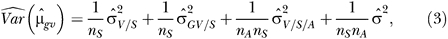


where *n_S_* = 2 and *n_A_* = 2.

Model II—one-sample/heteroscedastic errors: Model II is different from Model I by assuming that each gene has its own error distribution, so the model is fitted gene by gene. It can be represented by the following equation:


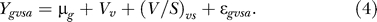


The parameter of interest in this model is *μ_gv_* = *μ_g_* + *V_v_,* which measures the mean of the SJ/IA indices of gene *g* in mutant *v*. The following null hypothesis defines the absence of DE in mutant *v* and gene *g*:


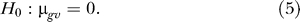


The variance of the treatment mean μ̂_*gv*_ can be computed by the following equation:


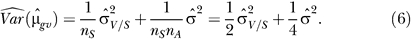


Model III—two-sample/homoscedastic errors: Model III differs from Model I by including the indices derived from the two wild-type self-hybridizations. Because of this inclusion, the study design is rendered unbalanced. To be more specific, the arrays in the two wild-type self-hybridizations came from the same sample, whereas the samples of four slides related to a mutant were from two distinct samples (see Figure 2B). The model can be represented by the following equation:


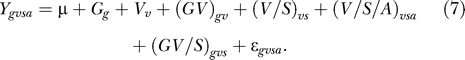


The parameter of interest in this model is *μ_gv_* = *μ* + *G_g_* + *V_v_* + *GV_gv_*, which measures the mean of the SJ/IA indices of gene *g* in mutant *v*. The following null hypothesis defines the absence of DE in mutant *v_m_* and gene *g* compared to the wild-type:


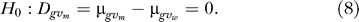


The variance of the treatment mean μ̂_*gv*_ can be computed by the following equation: 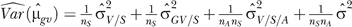 , where *n_S_* = 2 for mutants and *n_S_* = 1 for the wild-type.

Model IV—two-sample/heteroscedastic errors: Model IV differs from Model II by including the indices derived from the two wild-type self-hybridizations. The model can be represented by the following equation:


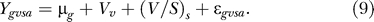


The parameter of interest in this model is *μ_gv_* = *μ_g_* + *V_v_,* which measures the mean of the SJ/IA indices of gene *g* in mutant *v*. The following null hypothesis defines the absence of DE in mutant *v_m_* and gene *g* compared to the wild-type:


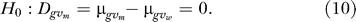


The variance of the treatment mean μ̂_*gv*_ can be computed by the following equation: 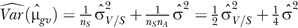 , where *n_S_* = 2 for mutants and *n_S_* = 1 for the wild-type.

### SHMM model.

Implementation of the SHMM model uses the R package EBarrays, available from ftp://ftp.biostat.wisc.edu/pub/newton/Arrays/tr1074/Rcode/. The output posterior probabilities for (directional) DE from the package have dual utilities: (i) ranking (genes), and (ii) calibration (providing FDR). We utilized the former for DEDS synthesis.

### DEDS procedures.

Fit the five DE models, and assume the resulting *p* values for gene *i* and model *j* are *p_ij_* (*i* = 1, 2, …, *n*, *j* = 1, 2, …, 5) in data matrix *P*.

Locate the most extreme point *E* as a vector of zeros of length five.

Calculate distance *d_i_* of all genes to *E* and order *d_(1)_* ≤ *d_(2)_* ≤ … ≤ *d_(n)_*.


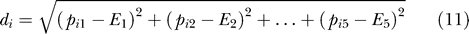


Generate *B* sets of reference distribution by:

Center the columns of *P* at mean 0.

Compute the singular value decomposition *P = UDV^T^*.

Calculate P* = PV.

Create Z* by drawing uniform distribution over the range of the columns of P*.

Back transform ***Z*** * by Z = Z*V^T^ to obtain the reference data *Z*.

For each reference dataset *b, d_i_* values are calculated and ordered in the way of


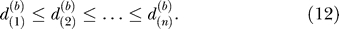


For a typical gene *i,* compute the median number of falsely called genes by computing the median number of values among each of the *B* sets of 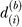 that are smaller than *d*_(*i*)_; and the *q*-value (controlling FDR) of gene *i* is computed as the median of the number of falsely called genes divided by the number of genes called significant. Illustration of determination of the extreme point *E* when using statistics (instead of *p*-values), in known null and non-null situations, is provided in Figure S1.

### Analysis of DE genes.

Gene annotations were obtained from the Ares lab intron database (http://www.cse.ucsc.edu/research/compbio/yeast_introns.html), and transcription frequency data was obtained from the Young lab (http://web.wi.mit.edu/young/expression/transcriptome.html).

The collection of all intron-containing genes was divided into sets of RP and non-RP genes, and averages and standard deviations were calculated for their transcription frequencies, intron lengths, and intron start sites. Several genes were omitted from these analyses because there was no good data concerning their transcription frequency or intron position or size. In addition, Mtr2, which has multiple, overlapping introns, was considered to have a single intron for this analysis (see Table S1). To determine if the properties of DE genes in a mutant were significantly different from those of all RP or non-RP intron-containing genes, we used a non-parametric resampling method. Briefly, a referent null distribution was generated by first taking 10,000 random samples of size *N* from the sets of all intron-containing RP or non-RP genes (*N* is the number of DE RP or non-RP genes for a particular mutant), and then calculating the averages of each sample. The *p-*value was derived as the percentage within the referent distribution that is more extreme than the observed property.

### QPCR analysis.

cDNA synthesis for QPCR was performed as described for fluorescently labeled target synthesis, except that equal concentrations of all four deoxyribonucleotides and no Cy dyes were used. Reactions lacking reverse transcriptase were performed to control for genomic DNA contamination. Amplifications were conducted in a Bio-Rad iCycler using iQ SYBR Green Supermix (Bio-Rad, Hercules, California, United States) and 200 μM primer according to the manufacturer's instructions, using the oligonucleotide primers found in Table S3. Representative transcripts were assayed in triplicate. To compare the QPCR with array values, we normalized QPCR values to the *OSH3* mRNA. *OSH3* was chosen as a suitable reference gene, since the array data indicated that its expression was unchanged in the five mutants used in the comparison.

## Supporting Information

Table S1 A Complete List of Differentially Expressed Genes for the Five Mutants in SJ and IA Indices (346 KB XLS)

Table S2 Comparison of the Five Models and DEDS in Terms of Numbers of False Positives/Negatives in the QPCR Tested Five Genes (23 KB DOC)

Table S3 Oligo Sequences Used in the QPCR Validation of the Microarray Analysis (14 KB XLS)

Figure S1 Illustration of DEDS Extreme Point Determination Application of DEDS in (a) the Affymetrix spike-in data ([45]); (b) the Affymetrix spike-in data with the top 100 DE genes removed to generate a dataset with only null genes. This dataset consists of 12,626 probe sets, 14 of which are spiked in at varying concentrations, and the rest are null. DEDS was applied synthesizing *t* statistics, fold change, and SAM (Significance Analysis for Microarrays) measures. The diagonal and upper triangle display Q-Q plots and scatterplots of the respective measures, while the lower triangle gives corresponding correlation coefficients. Red spots are differentially expressed by DEDS. *E* of (*t* statistic, fold change and SAM) was found to be (171.9, 7.2, 82.34) in panel (a) and at (7.5, 0.5, 3.1) in panel (b).
(39 KB DOC)

### Accession Numbers

The Saccharomyces Genome Database (SGD; http://www.yeastgenome.org/) accession numbers for the genes and gene products discussed in this paper are *ceg1* (S000003098), *RPL26A* (S000004336), *RPS25A* (S000003259), SNR17B (U3B) (S000007441), Spt4 (S000003295), Spt5 (S000004470), *U3B* (S000007441), YDR064W (RPS13) (S000002471), YGR027C (RPS25A) (S000003259), YLR344W (RPL26A) (S000004336), and YOL127W (RPL25) (S000005487).

## Abbreviations

ANOVA: analysis of variance
DE: differential expression
DEDS: Differential Expression via Distance Synthesis
df: degrees of freedom
IA: intron accumulation
QPCR: quantitative RT-PCR
RP: ribosomal
SHMM: semiparametric hierarchical mixture model
SJ: splice junction
