# Supplementary material for: Analysis of a Splice Array Experiment Elucidates Roles of Chromatin Elongation Factor Spt4–5 in Splicing
Source: PLoS Comput Biol. 2005 Sep 16;1(4):e39. doi: 10.1371/journal.pcbi.0010039 (PMC1214541; doi:10.1371/journal.pcbi.0010039)
Supplement: Supplementary file 1 [file pcbi.0010039.sg001.doc]

(a)

(b)

Figure S1: Application of DEDS in (a) the Affymetrix spike-in data (Irizarry et al., Nuclei Acids Research 2003 31(4): e15); (b) the Affymetrix spike-in data with the top 100 DE genes removed to generate a dataset with only null genes. The spikei-in dataset consists of 12626 probe sets, 14 of which are spiked-in at varying concentrations, and the rest are null. DEDS was applied synthesizing t-statistics, fold change and SAM measures. The diagonal and upper triangle display *Q-Q* plots and scatterplots of the respective measures while the lower triangle gives corresponding correlation coefficients. Red spots are DE by DEDS. *E* of (*t* statistic, fold change and SAM) was found to be (171.9, 7.2, 82.34) in panel (a) and at (7.5, 0.5, 3.1) in panel (b).
