# Supplementary material for: Analysis of a Splice Array Experiment Elucidates Roles of Chromatin Elongation Factor Spt4–5 in Splicing
Source: PLoS Comput Biol. 2005 Sep 16;1(4):e39. doi: 10.1371/journal.pcbi.0010039 (PMC1214541; doi:10.1371/journal.pcbi.0010039)
Supplement: Supplementary file 2 [file pcbi.0010039.st002.doc]

Table S3: Comparison of the 5 models and DEDS in terms of numbers of false positives/negatives over the QPCR tested 5 genes. Genes that are DE by the 5 models and DEDS have the *q* value ≤ 0.05. Concordance criterion is similar to Table 4 in which a gene is considered concordant between models and QPCR when 1) it is identified as non-DE by the models and its QPCR fold change is within the (-1, 1) thresholds; or 2) it is identify as DE by the models and its QPCR fold change is ≤ -1 or ≥ 1. Numbers of concordant genes for each method in each mutant are counted and displayed.

| MODEL | ***spt4Δ*** | ***spt5-4*** | ***spt5-242*** | ***spt5-194*** | ***ceg1-250*** |
| --- | --- | --- | --- | --- | --- |
| Model I | 2 | 4 | 1 | 3 | 4 |
| Model II | 2 | 2 | 0 | 3 | 4 |
| Model III | 2 | 1 | 1 | 2 | 3 |
| Model IV | 2 | 2 | 0 | 1 | 2 |
| Model V | 3 | 5 | 1 | 4 | 4 |
| DEDS | 3 | 5 | 1 | 4 | 4 |
